# Use of Wearable Sensors in Angelman Syndrome: A Systematic Review

**DOI:** 10.1111/jir.70121

**Published:** 2026-05-12

**Authors:** Veronika Vozka, Wei Siong Neo, Jane Kinkus Yatcilla, Carolyn E. B. McCormick, Roslyn Harold, Kimberly Gálvez‐Ortega, Mihir Dey, Laura E. Fitzpatrick, A. J. Schwichtenberg, Orlando S. Hoilett, Dan Foti, Bridgette L. Kelleher

**Affiliations:** ^1^ Department of Psychological Sciences Purdue University West Lafayette Indiana USA; ^2^ Libraries and School of Information Studies Purdue University West Lafayette Indiana USA; ^3^ Department of Human Development and Family Science Purdue University West Lafayette Indiana USA; ^4^ Department of Biomedical Engineering University of Cincinnati Cincinnati Ohio USA; ^5^ Weldon School of Biomedical Engineering Purdue University West Lafayette Indiana USA; ^6^ College of Engineering Purdue University West Lafayette Indiana USA

**Keywords:** Angelman, end point, genetic syndrome, outcome measures, personalized medicine, systematic review, wearables

## Abstract

**Background:**

Wearable sensors are a promising method for collecting clinical trial outcome data for people with Angelman syndrome (AS). However, there has yet to be a systematic probe into the ways in which wearable sensors have been successfully used in AS. The current study aims to provide a quantitative summary of wearable sensors used in AS, including contexts of use and psychometric properties, and to present key narrative highlights.

**Method:**

Literature searches were performed in three electronic databases: APA PsycInfo, PubMed and Web of Science Core Collection. Data items were categorized into four categories: sample characteristics, study methodological details, wearable sensor characteristics and psychometric properties assessed. Sample characteristics included sample size, age, biological sex, race/ethnicity and cognitive/developmental functioning. Study methodological details were subdivided into study design and setting. Wearable sensor characteristics included sensor type, placement site, means of attachment, assessed construct and sensor‐related data loss. Psychometric properties assessed included reliability and validity of sensor‐derived data.

**Results:**

We identified 16 articles through our systematic review. Wearable sensors were used to study sleep (*n* = 10, 62.5%), language (*n* = 2, 12.5%), gait (*n* = 2, 12.5%), caregiver proximity (*n* = 1, 6.3%), EEG power (*n =* 1, 6.3%), and arousal (*n* = 1, 6.3%) in AS through actigraphs, vocalization recorders, inertial sensors, radio‐frequency identification watches, wireless EEG caps, and functional near‐infrared spectroscopy caps, respectively. Findings from these studies broadly indicate that wearable sensors are feasible, reliable and valid for assessing a range of behaviours relevant to AS.

**Conclusions:**

Wearable sensors are a promising solution to enhance assessments in AS. However, with the small extant literature characterized by small sample sizes and restricted focus on a few relevant features in AS, there remains ample opportunities to explore the use of wearable sensors in people with AS. Additional studies will better inform clinical decision‐making and ultimately improve the lives of people with AS and their families.

Wearable sensors are body‐worn devices designed for continuous collection of behavioural and physiological data. They are a promising method for collecting clinical trial outcome data for a variety of clinical populations, including people with Angelman syndrome (AS). AS is a rare neurogenetic condition with a prevalence of approximately 1 in 15 000 births and is caused by deficient expression in neurons of the maternally inherited ubiquitin protein ligase E3A (*UBE3A*) gene located at 15q11‐q13 (Bird 2014). Four genetic mechanisms are known to result in AS: (a) deletion on the maternal 15q11‐q13 chromosome region (maternal deletion; 70%–75%); (b) inheritance of two paternal copies of the 15q11‐q13 chromosome segment (paternal uniparental disomy [UPD]; 1%–9%); (c) abnormality in the imprinting center of maternal chromosome 15 (imprinting defect; 1%–8%); and (d) *UBE3A* gene mutation on maternal chromosome 15 (mutation; 5%–10%; Bird 2014; Buiting et al. 2016). While there are some clinical phenotypic differences across these genotypes (Williams et al. 2010), AS is characterized by four consistent features: functionally severe intellectual disability, profound speech impairments, balance or movement disturbances and unique behavioural traits (e.g., frequent laughter or smiling; Bird 2014; Williams et al. 2006). Other clinical features that are frequently observed in more than 80% of individuals with AS include electroencephalography (EEG) abnormalities, epileptic seizures, gastrointestinal difficulties, microcephaly and sleep problems (Bird 2014; Esbensen and Schwichtenberg 2016; Williams et al. 2006).

Existing developmental and clinical measures are frequently inadequate or inappropriate for assessing various aspects of functioning in individuals with AS for several reasons. First, most assessment and outcome measures are not specifically designed or adapted for people with profound intellectual and multiple disabilities, which are common in AS (Soorya et al. 2018; Thurm et al. 2020). For example, attempts to use standardized tests in AS often reveal floor effects—minimum‐possible scores, despite variability in performance—impeding accurate and precise estimates of their functioning levels (DiStefano et al. 2020; Thurm et al. 2020). To circumvent floor effects, out‐of‐norm testing has been proposed, where assessments designed for individuals with younger chronological ages are used, though nontrivial limitations (e.g., plateaued scores and unintended stigmatizing effects) remain with such approaches (DiStefano et al. 2020).

A second limitation is current standardized assessment measures are not sufficiently sensitive for detecting acute, incremental changes, which are expected in individuals with AS given their slower developmental trajectories (Tan and Bird 2016). This lack of sensitivity is particularly problematic during clinical trials, which often aim to detect incremental gains in short periods of time. Clinical trials in AS increasingly aim to detect meaningful changes across domains such as epilepsy, sleep, motor function, communication and adaptive behaviour (Tjeertes et al. 2023). Emerging therapies may improve specific physiological and behavioural characteristics that affect daily functioning (Hagenaar et al. 2024). Therefore, outcome measures in AS trials must be sensitive to subtle changes and capable of capturing real‐world functioning. Many traditional clinical assessments rely on brief, structured observations that may not fully reflect day‐to‐day experiences or detect incremental change over time. Indeed, identifying suitable outcome measures that may serve as endpoints in clinical trials is one of the key unmet needs in AS clinical research (Markati et al. 2021). This need has become increasingly salient in the context of expanding natural history studies, deep phenotyping efforts and emerging gene‐targeted therapeutic approaches, including antisense oligonucleotide (ASO) strategies (Hagenaar et al. 2024; Markati et al. 2021). Importantly, parent‐ and caregiver‐identified priority domains for treatment‐related change include epilepsy, sleep, behaviour and communication, among others (Grieco et al. 2019; Wheeler et al. 2017). Together, these developments underscore the need for outcome measures that are sensitive to subtle change, scalable across time and capable of capturing real‐world functioning in domains that matter most to families and clinicians. Finally, existing assessment tools in AS rely heavily on informant report given substantial deficits in cognitive and expressive language abilities in AS. While caregiver reports are largely reliable and congruent with observations by clinicians (Tones et al. 2018), completing repeated assessments is time‐intensive, especially when used to monitor frequently occurring behaviours (e.g., challenging behaviours) and chronic challenges (e.g., sleep problems). Furthermore, informant reports are subject to biases and placebo effects (Bull et al. 2015) and, for these reasons, cannot serve as clinical endpoints for Food and Drug Administration trials. Caregivers also describe psychological challenges when asked to repeatedly report on their child's abilities, particularly when asked to report on weaknesses or complete forms that were not designed to reflect their child's unique skills (Walkowiak and Domaradzki 2025). Therefore, relying on subjective assessments alone is both incompatible with clinical trials in AS and potentially poses added burden for caregivers (Grieco et al. 2019; Wheeler et al. 2017).

## 1 Wearable Sensors as Potential Assessment Tools for AS

Wearable sensors may mitigate some of the challenges associated with traditional assessment tools (Park and Jayaraman 2021). For example, while most examiner‐facilitated assessment methods involve single observations of behaviours, wearable sensors allow for continuous monitoring (Dunn et al. 2018; Patel et al. 2012). This approach not only provides a greater volume of data but also enhances understanding of whether data patterns vary across different contexts, such as time of day or location. Wearable sensors also facilitate more naturalistic data collection, potentially revealing how an individual may experience their ‘typical’ environment. Naturalistic data have the potential to more accurately reflect the individual's real‐world experiences, particularly given common clinical comorbidities such as anxiety, which may compromise the accuracy of clinic‐based testing, particularly in unfamiliar environments (Edinger et al. 1997; Zhang et al. 2024). By reducing the need for in‐person assessments, wearable sensors also enhance accessibility, potentially laying the groundwork for more inclusive, representative science and clinical care (Pantelopoulos and Bourbakis 2010), so long as communities find the technology to be acceptable and appropriate (Dunn et al. 2018).

Despite these strengths, wearable sensors are not routinely used to collect clinical trial data in AS. Here, we focus on two fundamental barriers limiting translational outcomes. First, although wearable sensors are increasingly used to collect, process and integrate clinical data to reveal insights into activities and behaviours (Park and Jayaraman 2021), there has yet to be a systematic probe into the ways in which wearable sensors have been successfully used in AS. For example, depending on deployment characteristics and form factors (Park and Jayaraman 2021), some wearable sensors may have been more successfully used for measuring specific features of AS, whereas others may have received limited success due to poor tolerance and sensory‐compulsive behaviours (Grieco et al. 2019; Heald et al. 2020). Reviewing current wearable sensor use patterns in AS may therefore further guide patient‐centred and caregiver‐informed development and implementation of wearable sensors.

Second, there is limited information about the psychometric properties of wearable sensors currently used in AS. Rigorous psychometric evaluations of wearable sensors are critical (Sahin et al. 2019), especially because most commercially available products were not specifically designed for use in AS. Given that psychometric properties, such as reliability and validity, are sample‐specific, it is important to validate those properties in AS even if they had been shown to be satisfactory in non‐clinical samples during the technical development of wearable sensors. Additionally, examining the reliability and validity of wearable sensor data collected from individuals with AS is particularly important (Wasserman and Bracken 2013), given that the complex clinical profile (e.g., natural variation in behaviours and confounding influences of multiple symptoms) may affect these psychometric properties (Hagenaar et al. 2024). Similarly, sensitivity and feasibility are crucial wearable sensor attributes that need to be considered for effective clinical applications (Thurm et al. 2020). Specifically, emphasis may need to be placed on ensuring that these technologies are able to detect incremental changes, which may not be easily observed using existing behaviour‐based methodologies (Grieco et al. 2019). Acceptability is also important; sensors must be designed to support prolonged wearing by individuals with AS and be comfortable and practical for them and their families (Dunn et al. 2018).

The present study addressed these literature gaps by systematically reviewing published studies that used wearable sensors to measure and understand the experiences of individuals with AS. Across included studies, our primary objective was to provide a quantitative summary of wearable sensor details, including assessed psychometric properties and contexts in which they were used (i.e., sample and study characteristics). Our secondary aim was to provide a narrative synthesis of the included studies, highlighting key methodological approaches, wearable sensor characteristics and principal findings. Broadly, given the emerging use of wearable sensors in neurogenetic syndromes, we expected to find a limited number of studies that used wearable sensors in AS. We anticipated that existing studies would include technologies suited for assessing core clinical features of AS, including heart rate monitors (cognitive and attentional deficits), vocalization recorders (speech impairments), accelerometers and inertia sensors (sleep problems and movement disturbances) and EEG (neural abnormalities and seizures).

## 2 Methods

We report this systematic review according to guidelines specified in the Preferred Reporting Items for Systematic Reviews and Meta‐Analyses (PRISMA) statement (Page et al. 2021).

### 2.1 Eligibility Criteria

#### 2.1.1 Title and Abstract

Articles were retained for full‐text review if they met the following criteria: (1) The study included at least one participant with Angelman syndrome or a broader neurodevelopmental group that could plausibly include individuals with the condition; (2) the study used a wearable, body‐worn sensor for data collection; if it was unclear at this stage whether the device was untethered, the study was retained for full‐text review; (3) the study employed a design involving primary data collection (e.g., cross‐sectional, longitudinal, intervention); and (4) the publication was available in English and published after 1990. Studies were excluded at this stage if they clearly did not meet one or more of these criteria.

#### 2.1.2 Full Text

For a study to be included in this systematic review, all of the following inclusion criteria were met: (1) reported primary data (i.e., not secondary data analysis); (2) included at least one participant with a diagnosis of AS; (3) used at least one wearable sensor to objectively measure behavioural and/or physiological signals; (4) published in English as a peer‐reviewed journal article or peer‐reviewed conference paper; and (5) published after 1990. Studies were excluded if they did not meet all inclusion criteria. We restricted the publication date to facilitate targeted literature searches given that wearable technologies have been increasingly used for clinical purposes since the late 1990s (Pantelopoulos and Bourbakis 2010).

### 2.2 Information Sources

We performed literature searches in three electronic databases that focus on biomedical and psychological sciences: APA PsycInfo, PubMed and Web of Science Core Collection. Per one of the inclusion criteria, we narrowed literature searches to 1990 and onward. Searches were conducted in three phases on 12 October 2021, on 3 April 2024 and on 25 November 2025.

### 2.3 Search Strategies

Search strategies were jointly developed by clinical, engineering and information science faculty with expertise in AS and neurodevelopmental disorders, wearable technologies and systematic reviews, respectively. Full search strategies are presented in Supporting Information. Briefly, we used a combination of two search terms consisting of free‐text and controlled vocabularies. The first search strategy was used to identify studies involving AS (e.g., *“Angelman Syndrome”[Mesh]* or *“UBE3A”[Title/Abstract]*). The second search strategy was used to identify studies involving wearable sensors. We adopted a two‐pronged approach, differentiating sensors that have been specifically designed as wearables (e.g., *“Actigraphy”[Mesh]* or *smart glasses[Title/Abstract]*) from those that may be deployed as either fixed or portable systems; for the latter (e.g., *“Electrocardiography”[Mesh]* or *electroencephalogram[Title/Abstract]*), we used an additional search term to constrain sensors to those that are portable (e.g., *ambulatory[Title/Abstract]* or *untethered[Title/Abstract]*).

### 2.4 Selection Process

First, we uploaded study records retrieved from literature searches to Rayyan, an application specifically developed to support conducting systematic reviews (Ouzzani et al. 2016). Second, two undergraduate research assistants (hereafter ‘raters’) independently screened the title and abstract of each study record against inclusion criteria, with instructions to retain study records that provided limited details (e.g., participants described as having genetic disorders without specific mention of AS). In the event of disagreements, the raters reached consensus decisions through discussion under the supervision of a graduate‐level study member. Finally, after downloading full‐text articles, two raters independently conducted full‐text screening against inclusion criteria for each article. Discrepant decisions were resolved through discussion also under the supervision of a graduate‐level study member.

### 2.5 Data Items and Extraction Process

For each study included in this systematic review, two raters independently extracted data using a customized electronic form on REDCap (Harris et al. 2009). Discrepancies were resolved through discussion between the raters and, if necessary, in consultation with a lead graduate‐level study member with substantial clinical expertise in neurodevelopmental disorders and technical expertise using wearable sensors. Data items were categorized into four broad categories: sample characteristics, study methodological details, wearable sensor characteristics and the assessment of wearable sensor psychometric properties.

#### 2.5.1 Sample Characteristics

We coded *sample size* as the number of participants with AS who contributed to wearable sensor data. *Age* was coded in years using the statistics of mean, standard deviation and range. We coded *biological sex* as the percentage of participants with AS who were male. We coded *genotype* based on the four genetic aetiologies of AS (Bird 2014): maternal deletion; paternal UPD; imprinting defect; and mutation. *Race/ethnicity* were coded following federal racial and ethnic categories, including American Indian/Alaska Native, Asian, Black/African American, Hispanic/Latino, Native Hawaiian/Other Pacific Islander and White. We coded *cognitive or developmental functioning* using statistics of mean, standard deviation and range; we also coded which *assessment measure* was used.

#### 2.5.2 Study Methodological Details

We coded *study design* using three broad categories: intervention (i.e., study protocol included behavioural or pharmacological treatment for participants with AS); longitudinal (i.e., study protocol included multiple days of wearable sensor data collection); and methodological (i.e., study was primarily aimed at developing or evaluating methodologies for processing or analysing wearable sensor data). We also coded all *settings* where wearable sensor data were collected, using the categories of home, clinic/research laboratory and community site.

#### 2.5.3 Wearable Sensor Characteristics

Using prespecified options, we coded several wearable sensor characteristics, including sensor type, placement site, means of attachment, assessed construct and sensor‐related data loss. Here, for brevity, we provide brief definitions and illustrative examples for each of these characteristics. *Sensor type* refers to the type of wearable sensor used for measuring behavioural and/or physiological signals, such as accelerometer/actigraph, electroencephalograph, eye tracker, heart rate monitor and vocalization recorder. *Placement sites* are the physical body locations where the wearable sensor is worn, such as head, chest, waist, wrist and ankle. Wearable sensors may be affixed to their placement sites using various *means of attachment*, including adhesive, band, clip, clothing pocket and glove/sock. *Assessed construct* refers to the behavioural or psychological concept that the wearable sensor is primarily designed to measure, such as arousal, attention, gait, language and sleep. Sensor‐related *data loss* may occur due to several reasons, including poor data quality, technical failure and tolerance issues associated with sensory difficulties.

#### 2.5.4 Assessment of Wearable Sensor Psychometric Properties

We coded whether studies assessed the reliability and validity of wearable sensor data. Specifically, for *reliability*, we coded for interrater reliability (i.e., the degree to which two raters processed or interpreted the same wearable sensor data consistently) and test–retest reliability (i.e., the degree to which wearable‐derived measurements were consistent across two distinct assessment periods separated by time). In terms of *validity*, we broadly coded for convergent validity, based on whether studies compared wearable sensor data with other data assessing the same construct, such as behavioural observations/records, case–control comparisons, gold‐standard physiological assessments, questionnaires and rating scales.

## 3 Results

### 3.1 Search and Selection Results

**FIGURE 1:**
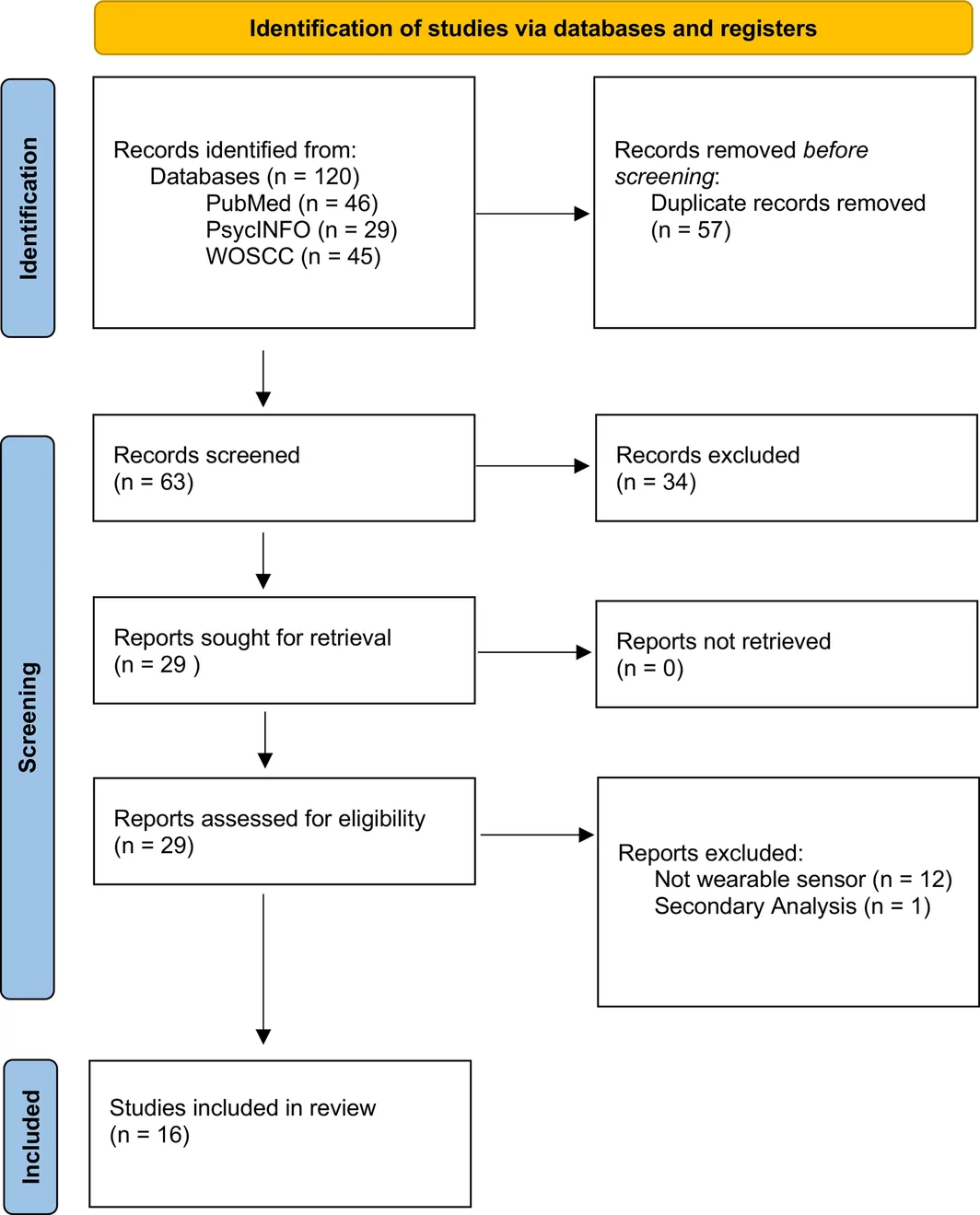
PRISMA flow diagram.

Our literature search and study selection results are depicted in Figure 1 as a PRISMA flow diagram. Briefly, literature searches yielded 63 study records, all of which were subject to title and abstract screening. Full‐text screening was completed for 29 study reports, of which 12 were excluded due to the use of non‐wearable sensors (e.g., polysomnography [Miano et al. 2005] and routine electroencephalogram [Robinson et al. 2015]) and one due to secondary data analysis (O'Sullivan et al. 2024). Therefore, this systematic review included a total of 16 studies (Tables 1 and 2; Agar et al. 2020; Agar et al. 2023; Allen et al. 2013; Anderson et al. 2008; Bindels‐de Heus et al. 2023; Duis et al. 2023; Gálvez‐Ortega et al. 2025; Goldman et al. 2012; Hagenaar et al. 2024; Hamrick et al. 2023; Iglesias Escalera et al. 2025; Kraan et al. 2022; Semenzin et al. 2021; Tjeertes et al. 2023; Trickett et al. 2019; Zhdanova et al. 1999). With the exception of the study by Zhdanova et al. (1999), all included studies were published in the last 10–15 years.

**TABLE 1:** Angelman syndrome sample characteristics and study methodological details.

Study	*n*	Age (years)	Sex (% male)	Genotype	Race/ethnicity	Cog/dev functioning	Study design	Study setting
Agar et al. 2020	12	*M* = 8.0 SD = 2.8 Range = 4.0–13.2	50.0	Unreported	Unreported	VABS‐II (ABC score) *M* = 47.0 SD = 7.6 Range = 32–57	Longitudinal (7 days)	Home
Agar et al. 2023	18	*M* = 9.3 SD = 3.2 Range = 4.5–15.1	38.9	Unreported	Unreported	Unreported	Longitudinal (3–6 days)	Home
Allen et al. 2013	5	*M* = 5.6 SD = 3.4 Range = 2.0–11.0	40.0	Unreported	Unreported	Unreported	Intervention (baseline: 2–9 weeks; intervention: 6–12 weeks; 1‐month follow‐up: 0–1 week; 3‐month follow‐up: 0–1 week)	Home
Anderson et al. 2008	3	*M* = 29.7 SD = 2.1 Range = 28.0–32.0	0.0	Maternal deletion (*n* = 3)	Unreported	Unreported	Longitudinal (14 days)	Home
Bindels‐de Heus et al. 2023	20	*M* = 7.5 SD = unreported Range = unreported	50.0	Maternal deletion (*n* = 7) Mutation (*n* = 7) Imprinting defect (*n* = 2) Paternal UPD (*n* = 4)	Unreported	Unreported	Intervention (baseline: 1 week; intervention: 6 weeks; 12‐week follow‐up: 1 week; 26‐week follow‐up: 1 week)	Home
Duis et al. 2023	23	*M* = 9.3 SD = 5.1 Range = 4.0–20.0	65.2	Maternal deletion (*n* = 14) Mutation (*n* = 4) Imprinting defect (*n* = 1) Paternal UPD (*n* = 4)	Unreported	Unreported	Longitudinal (14 days)	Home Community
Gálvez‐Ortega et al. 2025	7	*M* = 6.3 SD = 4.2 Range = 2.0–15.0	71.4	Unreported	White = 85.7% Hispanic/Latino = 14.3%	Unreported	Methodological	Home
Goldman et al. 2012	15	*M* = 6.5 SD = 4.4 Range = 2.0–16.0	40.0	Maternal deletion (*n* = 10) Mutation (*n* = 1) Imprinting defect (*n* = 1) Paternal UPD (*n* = 3)	Unreported	Unreported	Longitudinal (clinic: 1 day; home: 28 days)	Clinic Home
Hagenaar et al. 2024	28	*M* = 11.0 SD = 4.5 Range = unreported	50.0	Maternal deletion (*n* = 15) Mutation (*n* = 5) Imprinting defect (*n* = 1) Paternal UPD (*n* = 7)	Unreported	PEDI‐CAT *M =* 53.9 SD *=* 4.6 Range = unreported	Methodological	Clinic Community
Hamrick et al. 2023	27	*M* = 3.2 SD = 1.2 Range = 1.0–5.2	44.4	Maternal deletion (*n* = 21) Mutation (*n* = 1) Paternal UPD (*n* = 1) Unknown (*n* = 4)	White = 63.0% Multiple = 11.0% Unreported = 26.0%	VABS‐3 (ABC Score) *M* = 49.3 SD = 7.7 Range = 34–69	Methodological	Home
Iglesias Escalera et al. 2025	Treatment = 9 Control = 8	Treatment: *M* = 8.53 SD = 4.65 Range = unreported Control: *M* = 8.84 SD = 2.94 Range = unreported	Treatment: 44 Control: 75	Treatment: Maternal deletion (*n* = 5) Imprinting defect (*n* = 2) Duplication (*n* = 2) Control: Maternal deletion (*n* = 4) Imprinting defect (*n* = 0) Duplication (*n* = 4)	Unreported	Bayley III—Cognitive Treatment: *M =* 19.67 SD *=* 7.70 Range *=* unreported Control: *M =* 19.63 SD *=* 4.96 Range *=* unreported	Intervention (baseline: 5 days; intervention: 24 weeks)	Home
Kraan et al. 2022	5	*M* = 7.7 SD = 1.4 Range = 6.7–10.2	60.0	Maternal deletion (*n* = 1) Mutation (*n* = 3) Paternal UPD (*n* = 1)	Unreported	MSEL *M* = 28.2 SD = 3.7 Range = 23–33	Methodological	Home Clinic Community
Semenzin et al. 2021	10	*M* = 3.5 SD = unreported Range = 0.9–4.4	60.0	Unreported	Unreported	Unreported	Methodological	Home
Tjeertes et al. 2023	55	*M* = 10.3 SD = unreported Range = 1.0–38.0	56.4	Maternal deletion (*n* = 40) Mutation (*n* = 5) Imprinting defect (*n* = 4) Paternal UPD (*n* = 5) Unknown (*n* = 1)	Asian = 3.6% Black = 5.5% White = 85.5% Multiple = 5.5% Hispanic/Latino = 21.8%	Bayley‐III 1–4 years: *M =* 35.5 SD *=* 7.4 Range = 23–52 5–12 years: *M* = 49.9 SD = 11.1 Range = 29–79 ≥ 18 years: *M* = 50.9 SD = 9.6 Range = 30–66	Longitudinal (12 days)	Home
Trickett et al. 2019	20	*M* = 9.4 SD = 3.7 Range = unreported	40.0	Maternal deletion (*n* = 20)	Unreported	VABS‐II (standard Score) *M* = 44.0 SD = 9.2 Range = unreported	Longitudinal (7 days)	Home
Zhdanova et al. 1999	13	*M* = 6.5 SD = 2.8 Range = 2.0–10.0	30.8	Maternal deletion (*n* = 12) Paternal UPD (*n* = 1)	Unreported	Unreported	Intervention (baseline: 7 days; intervention: 5 days)	Home

Abbreviations: ABC = Adaptive Behavior Composite; Cog/dev functioning = cognitive/developmental functioning; MSEL = Mullen Scales of Early Learning; PEDI‐CAT = Pediatric Evaluation of Disability Inventory‐Computer Adaptive Test; UPD = uniparental disomy; VABS = Vineland Adaptive Behavior Scales.

**TABLE 2:** Wearable sensor characteristics and assessed psychometric properties.

Study	Type	Placement site	Attachment means	Assessed construct	Other relevant data	Data loss	Assessed psychometrics
Agar et al. 2020	Actigraph (Actiwatch 2)	Ankle, wrist	Band	Sleep	Sleep video recording	Unreported	Unreported
Agar et al. 2023	Actigraph (Actiwatch 2); RFID watch	Wrist; wrist	Band	Sleep; caregiver proximity	Sleep habits (FISH); sleep questionnaire (MSPSQ)	Unable to tolerate RFID watch overnight (*n =* 5)	Reliability (test–retest)
Allen et al. 2013	Actigraph (MicroMini Motionlogger)	Ankle	Band	Sleep	Sleep diary; Sleep questionnaire (CSHQ)	Unreported	Unreported
Anderson et al. 2008	Actigraph (Actiwatch)	Wrist	Band	Sleep	PSG; sleep diary	Unreported	Validity (comparison with sleep diary)
Bindels‐de Heus et al. 2023	Actigraph (MotionWatch)	Wrist	Band	Sleep	Sleep questionnaire (SPSQ)	Unable to tolerate Actiwatch (*n = 25*)	Reliability (test–retest)
Duis et al. 2023	Magneto‐inertial sensor (ActiMyo)	Ankle	Band	Gait	None	Unreported	Validity (comparison with gait deviation index)
Gálvez‐Ortega et al. 2025	EEG (ANT Neuro)	Head	Wireless cap	EEG power	Pre and post acceptability survey	Unable to schedule (*n = 1*)	Reliability (test–retest) Validity (comparison with typically developing children) Acceptability (post assessment ratings) Feasibility (session completion)
Goldman et al. 2012	Actigraph (Actiwatch AW‐64)	Wrist	Band	Sleep	PSG; sleep questionnaire (CSHQ)	Concurrent actigraphy data unavailable during PSG (*n* = 7); minimum actigraphy data unavailable (*n =* 4); parent assessed child would not tolerate wearing sensor (*n =* 1)	Reliability (test–retest); validity (comparison with PSG and sleep questionnaire)
Hagenaar et al. 2024	fNIRS (Brite 23)	Head	Wireless cap	Arousal	None	Study terminated due to tolerability issues and participants pulling off cap due to increased levels of distress	Unreported
Hamrick et al. 2023	Vocalization recorder (LENA)	Chest	Clothing pocket	Language/communication	Communication questionnaire (CSBS)	Unreported	Reliability (interrater); validity (comparison with communication measures)
Iglesias Escalera et al. 2025	Actigraph (Actiwatch 2)	Ankle	Band	Sleep	Sleep diary; sleep questionnaire (CSHQ)	Missing data occurred due to caregiver burden and device compliance issues (*n* = unreported)	Unreported
Kraan et al. 2022	Actigraph (Physiolog 5)	Ankle/foot	Shoe	Gait	None	Unreported	Reliability (test–retest)
Semenzin et al. 2021	Vocalization recorder (LENA)	Chest	Clothing pocket	Language/communication	None	Unreported	Reliability (interrater)
Tjeertes et al. 2023	Actigraph (GT9X Link)	Wrist, chest	Clothing pocket	Sleep	Sleep questionnaires (SNAKE and CSDI); sleep diary; seizure diary; sleep mat; PSG	Unable to tolerate device (*n =* 3); participant interference with device (*n =* 1); not feasible for caregiver (*n =* 3)	Validity (comparison with sleep questionnaire)
Trickett et al. 2019	Actigraph (Actiwatch 2)	Ankle, wrist	Band	Sleep	Sleep diary; sleep questionnaires (FISH, MESS, MSPSQ and PSQ)	Unable to tolerate wearing sensor (*n* = 2)	Validity (comparison with typically developing children)
Zhdanova et al. 1999	Actigraph (Mini‐logger 2000)	Back	Clothing pocket	Sleep	Sleep diary	Unreported	Validity (comparison with sleep diary)

Abbreviations: CSBS = Communication and Symbolic Behavior Scales; CSDI = Composite Sleep Disturbance Index; CSHQ = Children's Sleep Habits Questionnaire; EEG = electroencephalogram; FISH = Family Inventory of Sleep Habits; fNIRS = functional near infrared spectroscopy; MESS = Modified Epworth Sleepiness Scale; MSPSQ = Modified Simonds and Parraga Sleep Questionnaire; PSG = polysomnography; PSQ = Pediatric Sleep Questionnaire; RFID = radio‐frequency identification; SNAKE = Schlaffragebogen für Kinder mit Neurologischen und Anderen Komplexen Erkrankungen—Sleep Questionnaire for Children with Severe Psychomotor Impairment; SPSQ = Simonds and Parraga Sleep Questionnaire.

### 3.2 Descriptive Analyses

#### 3.2.1 Sample and Study Characteristics

Consistent with most research on rare neurogenetic syndromes, all included studies were characterized by small sample sizes, ranging from 3 to 55 participants (*M* = 16.4, SD = 12.6). Most studies (*n* = 15, 93.89%) focused on children. The weighted mean age across all studies was 8.4 years (range: 3.2–29.7). Biological sex was largely balanced, with 50% of all participants across studies being male. Among the 11 (68.8%) studies that reported genotype details, all four genetic aetiologies of AS were represented and broadly consistent with known distributions in natural history studies (maternal deletion: *n* = 152, 70%; paternal UPD: *n* = 26, 12%; imprinting defect: *n* = 11, 5.1%; mutation: *n* = 26, 12%). Race/ethnicity data were only reported in three studies (18.8%). Seven (43.8%) studies described participants' cognitive/developmental functioning using standardized instruments. Of these studies, two (12.5%) used the Vineland‐II, two (12.5%) used the Vineland‐3, two (12.5%) used the Bayley Scales of Infant and Toddler Development, one (6.3%) used the Mullen Scales of Early Learning and one (6.3%) used the Pediatric Evaluation of Disability Inventory Computer Adaptive Test.

A mix of study designs was utilized, with most studies (*n* = 11, 68.8%) collecting wearable sensor data over multiple days (range: 3 days–24 weeks) as part of longitudinal or intervention protocols. Most included studies (*n =* 14, 87.5%) primarily collected wearable sensor data in the home setting.

#### 3.2.2 Wearable Sensors

Wearable sensors were used to study sleep (*n* = 10, 62.5%), language (*n* = 2, 12.5%), gait (*n* = 2, 12.5%), caregiver proximity (*n* = 1, 6.3%), EEG power (*n* = 1, 6.3%) and arousal (*n* = 1, 6.3%) in AS through accelerometers/actigraphs, vocalization recorders, inertial sensors, radio‐frequency identification (RFID) watches, wireless EEG caps and functional near‐infrared spectroscopy (fNIRS) caps, respectively. These sensors were generally worn as designed, such as actigraphs being attached to the ankle or wrist using a band and vocalization recorders being placed in the front chest pocket of a vest. Half of the studies (*n* = 8, 50.0%) did not report information about wearable sensor data loss. In the remaining eight studies with information about data loss, six reported that a small number of participants could not tolerate wearing an actigraph; one study decided to halt the use of fNIRS entirely due to the distress displayed by participants when wearing an fNIRS cap. In terms of assessed psychometric properties, seven (43.8%) studies examined test–retest or interrater reliability, eight (50.3%) studies investigated convergent validity using varied methodologies, including behavioural records and questionnaires, and one study (6.3%) used post‐assessment survey to measure acceptability.

### 3.3 Narrative Reviews

Here, we summarize key study findings with a specific emphasis on wearable device methodology, psychometric analyses and key clinical findings. Studies utilized wearable devices in both children (e.g., Goldman et al. 2012; Trickett et al. 2019) and adults (e.g., Anderson et al. 2008) with AS using several study designs.

#### 3.3.1 Sleep

##### 3.3.1.1 Feasibility Study

All included studies that examined sleep in AS used actigraphy. Tjeertes et al. (2023) compared several different methods of collecting sleep data in individuals with AS, including a sleep diary, a sleep mat, actigraphy and overnight EEG. Daily adherence and overall caregiver satisfaction with each method were assessed. Mean adherence to the actigraph in participants with AS was 56%, with lack of feasibility for caregivers, inability of participants to tolerate the device and participants interfering with the device being reasons given for nonadherence. Caregivers were more satisfied with actigraphy than overnight EEG (64% vs. 59% were satisfied or very satisfied) but were most satisfied with the sleep mat (91% were satisfied or very satisfied).

##### 3.3.1.2 Sleep Characterization Studies

Eight studies used actigraphy to objectively characterize sleep behaviours. Using a case‐study approach, Anderson et al. (2008) examined sleep disturbances in three sisters using actigraphy and caregiver‐report sleep diaries. Some discrepancies emerged across methods for total sleep time, with caregivers reporting 9–10 h of sleep in sleep diaries and actigraph data indicating 7–8.5 h of sleep. Furthermore, actigraphy yielded additional sleep metrics, including low sleep efficiency and high sleep fragmentation, that were not detectable by caregiver diary methods.

Using both objective (i.e., actigraphy and polysomnography) and subjective (i.e., parent‐report questionnaire) sleep measures, Goldman et al. (2012) examined sleep behaviours in children with AS and their associations with parental stress. Actigraph‐determined night wakings were similar across clinic and home settings (Spearman's *r*_*s*_ = 0.76), providing some support for reliability of repeated actigraph measurements across settings. Additionally, night wakings detected via home‐based actigraphy were similar to those detected using gold‐standard clinical polysomnography (*r*_*s*_ = 0.64), indicating reasonable convergent validity. Participants with longer sleep duration, as measured by actigraphy, also exhibited greater parent‐reported night wakening and parasomnia, which was suggested to reflect how extended sleeping hours are needed to compensate for sleep disruptions in AS.

Trickett et al. (2019) used a case–control design to contrast sleep parameters for children with AS with those for age‐ and sex‐matched typically developing (TD) children, leveraging actigraphy and parent‐reported sleep diaries. Compared to controls, children with AS exhibited earlier ‘lights out’ time (Cohen's *d* =0.47) and poorer sleep efficiency (*d* = 0.33). Additionally, children with AS exhibited greater intra‐ and inter‐individual variability in total sleep time and night wakening relative to TD children.

Agar et al. (2020) used actigraphy to examine settling—the period between bedtime and the onset of sustained sleep—and night awakenings in children with AS and Smith‐Magenis syndrome. The most common behaviours demonstrated by children with AS during these awake periods included mouthing, interacting with toys and electronic devices, expressing negative facial expressions and vocalizations and rocking. Additionally, lag analyses revealed that pain‐related behaviours (e.g., specific leg movements and negative affect) were more likely to occur before night awakenings for children with AS, indicating that pain and discomfort may be a cause of sleep problems in AS.

In a later study, Agar et al. (2023) investigated night wakings with a focus on overnight parent–child interactions, using RFID watches to track child‐caregiver proximity and actigraphy to define sleep–wake periods. Findings indicated that children with AS experienced poor sleep quality and prolonged night wakings regardless of the frequency of overnight caregiver interactions, suggesting that insomnia in AS cannot be explained solely by operant models.

##### 3.3.1.3 Intervention Studies

Two studies evaluated behavioural interventions, one study evaluated a pharmacological intervention and one study evaluated a dietary intervention. To assess the effectiveness of a parent‐led behavioural treatment package on reducing chronic sleep problems, Allen et al. (2013) used a multiple baseline design on five children with AS, where actigraph data were collected during baseline, treatment and follow‐up periods. Actigraphy indicated an increase in total sleep time, a slight increase in sleep efficiency, a slight reduction in latency to fall asleep and unchanged duration of night wakening, suggesting that the behavioural treatment package successfully improved specific aspects of sleep in AS.

Bindels‐de Heus et al. (2023) used actigraphy and videosomnography to measure the effects of a behavioural treatment program on sleep quality in children with AS. Results showed an improvement in total sleep time and wake after sleep onset following the intervention, as well as generally enhanced quality of life measures for both children and caregivers, providing evidence for the effectiveness of behavioural intervention programs at treating sleep difficulties associated with AS. Notably, actigraphy underestimated total sleep duration when compared to videosomnography due to movements during sleep being mischaracterized as waking by the actigraph.

Zhdanova et al. (1999) used actigraphy to monitor the effects of melatonin therapy on sleep behaviours in children with AS who were experiencing insomnia. Melatonin treatment resulted in a significant increase in total sleep time, which was also accompanied by a significant reduction in motor activity during sleep. Importantly, these positive effects measured by actigraphy were consistent with parent reports of improved sleep quality, providing converging evidence of the benefits of low‐dose melatonin therapy for AS.

Iglesias Escalera et al. (2025) used actigraphy to assess sleep quality in response to a low‐glycemic‐index ketogenic diet in children with AS. Sleep parameters assessed included sleep latency, number of awakenings, sleep efficiency, and wake time after sleep onset. Results indicated a minor increase in sleep duration and small changes in other actigraphy‐derived sleep parameters, consistent with Children's Sleep Habits Questionnaire findings, with no statistically significant differences between the treatment and control groups. Overall, actigraphy findings were consistent with prior literature documenting persistent and significant sleep disturbances in individuals with AS.

#### 3.3.2 Language

Wearable vocalization recorders, such as the Language Environment Analysis (LENA) system used by Semenzin et al. (2021) and Hamrick et al. (2023), facilitate large‐scale collection of vocalization data in naturalistic settings. Semenzin et al. (2021) evaluated a crowdsourcing‐based annotation pipeline involving citizen scientists, where they examined whether nonexpert annotators could reliably categorize vocalizations into five categories (i.e., canonical, noncanonical, crying, laughing and junk), which were subsequently used to derive two composite metrics (i.e., linguistic proportion and canonical proportion). Although interrater reliability for individual vocalizations was only moderately strong, reliability was considerably higher for composite metrics.

Leveraging an overlapping, expanded sample to Semenzin et al. (2021), Hamrick et al. (2023) examined how large‐scale vocalization data collected in naturalistic settings could be enhanced through semi‐automated processing systems. Specifically, Hamrick et al. (2023) developed an utterance‐level processing (ULP) system to improve upon LENA's automated output by incorporating strategic human coding to generate granular vocal maturity metrics (e.g., canonical babbling ratio and canonical vocalization rate), which are particularly relevant for quantifying language in preverbal children with AS. Their work demonstrated that ULP‐derived metrics could be reliably coded and provided developmentally meaningful information in both low‐risk and AS samples.

Together, these two studies illustrate complementary strategies for scaling annotation of naturalistic vocalization data in AS: Hamrick et al. (2023) highlights the value of structured semi‐automated pipelines for clinical‐grade measurements, while Semenzin et al. (2021) shows the promise of crowdsourced approaches to improve scalability in research applications.

#### 3.3.3 Gait Analysis

Two studies performed gait analysis using wearable sensors in individuals with AS, focusing on both feasibility and clinical relevance. Feasibility was first established by Kraan et al. (2022), where an ankle‐worn sensor was used to collect real‐world walking data across short and long walks in multiple settings (i.e., home, research centre and hospital). Measured parameters included mean stride length, mean stance percentage and coefficient of variation for stance percentage. Notably, these metrics could be reliably collected and showed high agreement with laboratory‐based assessments.

Duis et al. (2023) built on Kraan et al. (2022) by employing the ActiMyo magneto‐inertial sensor to assess stride length, stride velocity and stride duration in a larger sample of participants with AS and compared their data to TD controls. Individuals with AS exhibited significantly shorter stride length, reduced gait speed and wider strides with shorter steps, with these features being consistent across age groups.

Together, these two studies support the feasibility of wearable gait analysis in AS and suggest that wearable‐derived metrics are sensitive to core motor abnormalities. However, larger samples and longitudinal designs are still needed to determine their utility as clinical endpoints or markers of change over time.

#### 3.3.4 Cortical Activity

fNIRS is a method to measure cortical activity, which is passive, noninvasive and does not require participants to remain still. Hagenaar et al. (2024) assessed the use of a wearable sensor for measuring cortical activity as part of a feasibility study that examined the tolerability of various monitoring systems in AS (e.g., eye tracking and body‐impedance analysis). Specifically, an untethered fNIRS cap was used for measuring cortical activation in response to social versus non‐social stimuli in children with AS. However, no conclusions about cortical activation could be drawn due to significant tolerability issues that was causing distress in some participants. Of the 20 participants for whom fNIRS measurements were attempted, nearly half (*n* = 9) were unable to complete the measurement due to distress caused by the wearable sensor; even for those who successfully completed the measurement, they were too distracted by the wearable sensor to pay attention to presented stimuli. Only a single participant had fNIRS data of sufficient quality for analysis, suggesting that current fNIRS technology may not be feasible for individuals with AS while concurrently highlighting significant opportunities for further research and development of fNIRS technology in AS.

#### 3.3.5 Electroencephalography (EEG) Delta Power

EEG records brain electrical activity, with elevated delta power serving as a key biomarker of cognitive function and treatment response in Angelman syndrome. Gálvez‐Ortega et al. (2025) evaluated the feasibility, acceptability, reliability and validity of a remote EEG assessment. Delta power was measured using a wearable EEG cap with dry electrodes during a resting state task involving a silent video of floating geometric fractals. In seven children with AS, the protocol demonstrated high completion rates and satisfaction (91%), supporting acceptability. Reliability was supported by internal consistency ratings and test–retest measures, and validity was established by expected group differences in delta power between AS control participants consistent with previous research. Overall, these findings highlight the importance of patient‐centred design for successful implementation of remote EEG protocols in sensory‐challenging populations.

## 4 Discussion

Wearable sensors are a promising solution to enhance assessments in AS. We identified 16 articles through our systematic review, indicating substantial opportunities to continue exploring the use of wearable sensors in AS. The current literature suggests that some signals of interest in AS could be successfully monitored using wearable sensors across naturalistic settings, including sleep, language, EEG power and gait. Notably, these measurements showed significant associations with validated assessment tools, such as caregiver reports and clinical polysomnograms. Additionally, some of the reviewed studies were successfully implemented in the home setting, which presents significant advantages over lab‐based systems that typically tether participants and involve protocols outside of normal daily routines, which may contribute to confounding variables that affect the collection and interpretation of behavioural and physiological data. These findings have promising implications for clinical trials, particularly in the context of costly gene therapy interventions in AS. Wearable sensors enable continuous, objective monitoring in real‐world environments and may generate digital biomarkers capable of detecting subtle treatment‐related changes that clinical standardized assessments might overlook. In current trials, wearable‐derived measures may be best positioned as complementary or exploratory endpoints alongside established clinical assessments while further validation work continues. As such, they may enhance sensitivity to change while potentially reducing participant burden and improving trial efficiency. Despite this promise, the current literature remains limited. With only a small number of studies (*n* = 16), small sample sizes (*n* = 55 or less participants) and limited assessment of features relevant to AS (sleep, language, gait, EEG power), there remains ample opportunities to explore the use of wearables in people with AS.

Despite the limited literature identified in this study, our review generally supports the notion that wearable sensor use is feasible in AS; wearable sensors have been successfully used to track sleep patterns, capture language‐related vocalizations and quantify movement and gait abnormalities across real‐world settings. Several sensors were reliable across settings (Agar et al. 2023; Bindels‐de Heus et al. 2023; Hamrick et al. 2023; Kraan et al. 2022; Semenzin et al. 2021), valid against gold‐standards (Duis et al. 2023; Goldman et al. 2012; Hamrick et al. 2023; Tjeertes et al. 2023), sensitive to the AS phenotype (Duis et al. 2023; Goldman et al. 2012; Hamrick et al. 2023; Kraan et al. 2022; Tjeertes et al. 2023; Gálvez‐Ortega et al. 2025) and feasible with limited missing data (Agar et al. 2020; Agar et al. 2023; Goldman et al. 2012; Tjeertes et al. 2023; Trickett et al. 2019; Zhdanova et al. 1999; Gálvez‐Ortega et al. 2025). For example, Goldman et al. (2012) reported actigraphy‐derived sleep data corresponded with clinical polysomnogram recordings and Hamrick et al. (2023) demonstrated expressive language signals were reliably captured through wearable audio recorders. Given the importance of measuring these constructs in clinical trials, these preliminary gains represent substantial field advances for the AS community.

Across studies, we noticed a limited number of form factors used in AS wearable sensor research, relative to the types of technologies being developed in the field. We noted studies primarily used wristwatches, anklets and pocket recorders. These limited form factors inherently constrain the specific sensing modalities that can be achieved. Given wearables have more extensive form factors such as shirts (Elliot et al. 2019; Smith et al. 2019), vests (Islam et al. 2024), undergarments (Lee et al. 2023) and more (Bluhm et al. 2024; Charlton et al. 2023; Davis et al. 2024; Kim et al. 2017), there are immense opportunities to explore different types of wearables with the AS population. Furthermore, it is possible that processing behavioural and physiological data collected from individuals with AS poses more challenges than those from neurotypical individuals. It is well established that physiological signals, such as electrocardiography, EEG and respiration, require significant post‐processing in order to extract useful clinical metrics (Bent and Dunn 2020; Bent et al. 2020; Chao et al. 2022; Hoilett et al. 2023; Kim et al. 2017; Pan and Tompkins 1985); given differences in underlying physiological functions in AS (e.g., differences in percent power and spike activity in EEG), additional challenges in data processing may be present for AS and remain to be further explored.

One of the most significant opportunities for further research is to expand the sensing capabilities within the AS population using wearable devices. With assessments currently limited to specific aspects of sleep, language and gait, other outcome measures such as reducing seizures and improving behavioural problems have not been prioritized to be assessed using wearable sensors. For example, EEG is critical for quantifying sleep disturbances more optimally, including seizures and sleep quality (den Bakker et al. 2018; Ewen et al. 2019; Laan and Vein 2005). Heart rate is correlated with stress, anxiety and general health and fitness, which may be leveraged to assess behavioural and physiological differences in individuals with AS, similar to existing approaches used with other neurodevelopmental disorders such as autism (Kelleher et al. 2020; Kim et al. 2018; Neo and Kelleher 2021; Perrot et al. 2021). Therefore, given the initial success, many studies are reporting for using sensors in AS, exploring additional sensing capabilities may improve our understanding of AS phenotypes and support needs.

Our systematic review indicated that no studies had used head‐mounted cameras in their work with individuals with AS. Head‐mounted cameras are widely used in research with neurodevelopmental populations, including autism, Down syndrome and Prader‐Willi syndrome (Bauer et al. 2023; Newbutt et al. 2020; Perkovich et al. 2024; Yurkovic et al. 2021), and may similarly provide critical behavioural insights for individuals with AS. Furthermore, we only found two studies that used vocalization recorders to examine communication features in AS (Hamrick et al. 2023; Semenzin et al. 2021). These observations are somewhat surprising, considering the enormous body of existing literature that focuses on understanding behavioural and psychological constructs in neurodevelopmental conditions using audio‐visual data, such as communication, interpersonal interactions and stress. It is plausible that the limited use of audio‐visual data streams in AS may reflect barriers in implementing these technologies, and additional technical development work is needed.

Relatedly, some of our reviewed studies reported that some participants were unable to tolerate wearing the biosensors (Agar et al. 2023; Bindels‐de Heus et al. 2023; Goldman et al. 2012; Hagenaar et al. 2024; Tjeertes et al. 2023; Trickett et al. 2019; Iglesias Escalera et al. 2025). These difficulties suggest a need for designing wearables that accommodate sensory sensitivities and needs frequently documented in AS. One possibility is to incorporate e‐textiles with different fabric compositions and textures that prioritize comfort and meet the unique biometric needs of the AS population (Elliot et al. 2019; Islam et al. 2024; Kim et al. 2017; Smith et al. 2019). The lack of widespread use of wearable sensors in the AS community may indicate that currently available wearables do not adequately address the needs of neurodevelopmental populations, an issue that could be addressed using patient‐informed designs gathered through focus groups and caregiver surveys. As the development of wearable sensors for these populations expands, it will also be important to standardize reporting standards to ease cross‐study comparisons (APA Publications and Communications Board Working Group on Journal Article Reporting Standards 2008). Several limitations should be considered. First, our review was restricted to English‐language publications that may have excluded relevant international studies. Second, only peer‐reviewed publications indexed in selected databases were included, and thus, our review may not capture early stage studies often published in conference proceedings or engineering venues. Third, given the rapid pace of technological development, findings from earlier studies may no longer reflect current device capabilities. Fourth, a formal methodological quality assessment was not conducted in this review. While this approach is not fully aligned with PRISMA risk of bias recommendations, it reflects the primary objective of this study, which was to provide a descriptive overview of wearable sensor use in AS rather than to evaluate or synthesize intervention effects. As a result, the findings should be interpreted with caution, as the included studies may vary in methodological rigour and potential bias. Future reviews aiming to evaluate effectiveness or compare outcomes would benefit from incorporating formal quality assessment.

## 5 Conclusion

Use of wearable sensors in AS is severely limited, though the few studies our systematic review uncovered did report some positive experiences and outcomes. In summary, there are immense opportunities to further explore the utility of wearable sensors in AS research and clinical practice, which may ultimately improve the lives of people with AS and their families. However, given the small number of published studies to date and reported challenges in effectively engaging individuals with AS, future work should actively incorporate community perspectives to ensure that advancements in the use of wearable sensors in research and clinical spaces align with the most pressing needs of the AS community.

## Funding

This project was supported by an internal grant from the Purdue Institute for Integrative Neuroscience (PIIN; MPI Kelleher/McCormick). Orlando S. Hoilett was supported by PIIN and the College of Engineering. Laura E. Fitzpatrick was supported in part by the University of Cincinnati College of Engineering and Applied Science Protégé Undergraduate Research Program and the University of Cincinnati College of Engineering and Applied Science Undergraduate Research Co‐op Fellowship Program. Mihir Dey was supported in part by the University of Cincinnati Undergraduates Pursuing Research in Science and Engineering Program.

## Conflicts of Interest

Orlando S. Hoilett is a co‐founder of Predictive Wear Inc. Orlando S. Hoilett is a co‐inventor on patents US11672288B2 and US12063993B2 issued to Predictive Wear Inc. Orlando S. Hoilett is a co‐inventor on patents US20230263248A1 and US20210052026A1 pending to Predictive Wear Inc. Orlando S. Hoilett is a co‐inventor on patents US11737708B2, US11633152B2, US12029548B2, US11737708B2, US10786201B2, US11090649B2, US11628434B2 and US11529626B2 issued to Purdue Research Foundation. Orlando S. Hoilett is a co‐inventor on patents WO2020009798A1, US20240000384A1 and WO2017184665A1 pending to Purdue Research Foundation. Bridgette Kelleher is a paid consultant for work unrelated to this project.

## Supporting information


**Data S1:** Supporting information.

## Data Availability

The authors have nothing to report.
