# Supplementary material for: Use of Wearable Sensors in Angelman Syndrome: A Systematic Review
Source: J Intellect Disabil Res. 2026 May 12;70(10):985–1000. doi: 10.1111/jir.70121 (PMC13553319; doi:10.1111/jir.70121)
Supplement: Supplementary file 1 [file JIR-70-985-s001.docx]

**Supplementary Materials**

# Full Search Strategies

Full search strategies for individual electronic databases are detailed below.

***APA PsycInfo***

S1. TI ( ambulatory OR bracelet* OR external OR “head mounted” OR headset* OR “head set*” OR “home based” OR “home environment” OR mobile OR “natural environment” OR naturalistic OR personal OR “point of care” OR portable OR remote OR “skin mounted” OR noninvasive OR “non invasive” OR smart OR tetherless OR untethered OR wearable OR wireless* OR “wrist mounted” ) OR AB ( ambulatory OR bracelet* OR external OR “head mounted” OR headset* OR “head set*” OR “home based” OR “home environment” OR mobile OR “natural environment” OR naturalistic OR personal OR “point of care” OR portable OR remote OR “skin mounted” OR noninvasive OR “non invasive” OR smart OR tetherless OR untethered OR wearable OR wireless* OR “wrist mounted” ) OR SU ( ambulatory OR bracelet* OR external OR “head mounted” OR headset* OR “head set*” OR “home based” OR “home environment” OR mobile OR “natural environment” OR naturalistic OR personal OR “point of care” OR portable OR remote OR “skin mounted” OR noninvasive OR “non invasive” OR smart OR tetherless OR untethered OR wearable OR wireless* OR “wrist mounted” )
S2. DE (Electrocardiography OR Electroencephalography OR Galvanic Skin Response ) OR TI ( biosensor* OR “brain monitor*” OR device* OR ECG OR EEG OR electrocardiography OR electrocardiogram OR electrodermal OR electroencephalogram OR electroencephalography OR FIRS OR functional near infrared spectroscopy OR "heart monitor*" OR sensor* OR “skin conductance” ) OR AB ( biosensor* OR “brain monitor*” OR device* OR ECG OR EEG OR electrocardiography OR electrocardiogram OR electrodermal OR electroencephalogram OR electroencephalography OR FNIRS OR functional near infrared spectroscopy OR "heart monitor*" OR sensor* OR “skin conductance” ) OR SU ( biosensor* OR “brain monitor*” OR device* OR ECG OR EEG OR electrocardiography OR electrocardiogram OR electrodermal OR electroencephalogram OR electroencephalography OR FNIRS OR functional near infrared spectroscopy OR "heart monitor*" OR sensor* OR “skin conductance” )
S3. S1 AND S2
S4. DE ( "Actigraphy" OR "Hearing Aids" OR OR "Polysomnography" OR Smartphones OR "Speech and Hearing Measures" OR "Wearable Devices" ) OR ( TI ( accelerometry OR accelerometer* OR actigraph* OR actigram OR electropalatograph* OR electroglottography OR “eye movement tracking” OR “eye tracking” OR “glottal waveform*” OR gyroscope* OR “hearing aid*” OR “hearing test*” OR inertia OR inertial OR “language environment analysis” OR “motion sensor*” OR “motion sensing” OR “nasal oral ratio*” OR nasometry OR nasality OR nasalance OR “physiological arousal” OR polysomnography OR polysomnographies OR “sleep monitor*” OR “smart bandage*” OR “smart device*” OR “smart glasses” OR “smart phone*” OR smartglasses OR smartphone* OR somnography OR somnographies OR sonography OR sonographies OR “sound spectrogram*” OR “vocalization recording*”) OR AB ( accelerometry OR accelerometer* OR actigraph* OR actigram OR electropalatograph* OR electroglottography OR “eye movement tracking” OR “eye tracking” OR “glottal waveform*” OR gyroscope* OR “hearing aid*” OR “hearing test*” OR inertia OR inertial OR “language environment analysis” OR “motion sensor*” OR “motion sensing” OR “nasal oral ratio*” OR nasometry OR nasality OR nasalance OR “physiological arousal” OR polysomnography OR polysomnographies OR “sleep monitor*” OR “smart bandage*” OR “smart device*” OR “smart glasses” OR “smart phone*” OR smartglasses OR smartphone* OR somnography OR somnographies OR sonography OR sonographies OR “sound spectrogram*” OR “vocalization recording*” ) OR SU (accelerometry OR accelerometer* OR actigraph* OR actigram OR electropalatograph* OR electroglottography OR “eye movement tracking” OR “eye tracking” OR “glottal waveform*” OR gyroscope* OR “hearing aid*” OR “hearing test*” OR inertia OR inertial OR “language environment analysis” OR “motion sensor*” OR “motion sensing” OR “nasal oral ratio*” OR nasometry OR nasality OR nasalance OR “physiological arousal” OR polysomnography OR polysomnographies OR “sleep monitor*” OR “smart bandage*” OR “smart device*” OR “smart glasses” OR “smart phone*” OR smartglasses OR smartphone* OR somnography OR somnographies OR sonography OR sonographies OR “sound spectrogram*” OR “vocalization recording*” ) )
S5. S3 OR S4
S6. ( (TI ( "angelman syndrome" OR "angelman's syndrome" OR "happy puppet" OR UBE3A ) OR AB ( "angelman syndrome" OR "angelman's syndrome" OR "happy puppet" OR UBE3A ) OR SU ("angelman syndrome" OR "angelman's syndrome" OR "happy puppet" OR UBE3A ) )
S7. S6 AND S5
S8. S7 AND PY=1990-2024
S9. S8 AND Language=English

***PubMed***

S1. "Angelman Syndrome"[Mesh] OR "angelman syndrome"[Title/Abstract] OR "angelman's syndrome"[Title/Abstract] OR "happy puppet"[Title/Abstract] OR UBE3A[Title/Abstract]
S2. "Accelerometry"[Mesh] "Actigraphy"[Mesh] OR "Eye-Tracking Technology"[Mesh] OR "Glottis/diagnostic imaging"[Mesh] OR "Hearing Aids"[Mesh] OR “Hearing Tests”[Mesh] OR "Monitoring, Physiologic"[Mesh] OR "Polysomnography"[Mesh] OR "Smart Glasses"[Mesh] OR "Sound Spectrography"[Mesh] OR "Wearable Electronic Devices"[Mesh]
S3. accelerometry[Title/Abstract] OR accelerometer*[Title/Abstract] OR actigraph*[Title/Abstract] OR actigram[Title/Abstract] OR electropalatograph*[Title/Abstract] OR electroglottography[Title/Abstract] OR eye movement tracking[Title/Abstract] OR eye tracking[Title/Abstract] OR glottal waveform*[Title/Abstract] OR gyroscope*[Title/Abstract] OR hearing aid*[Title/Abstract] OR hearing test*[Title/Abstract] OR inertia[Title/Abstract] OR inertial[Title/Abstract] OR language environment analysis[Title/Abstract] OR motion sensor*[Title/Abstract] OR motion sensing[Title/Abstract] OR nasal oral ratio*[Title/Abstract] OR nasometry[Title/Abstract] OR nasality[Title/Abstract] OR nasalance[Title/Abstract] OR physiological arousal[Title/Abstract] OR polysomnography[Title/Abstract] OR polysomnographies[Title/Abstract] OR sleep monitor*[Title/Abstract] OR smart bandage*[Title/Abstract] OR smart device*[Title/Abstract] OR smart glasses[Title/Abstract] OR smart phone*[Title/Abstract] OR smartglasses[Title/Abstract] OR smartphone*[Title/Abstract] OR somnography[Title/Abstract] OR somnographies[Title/Abstract] OR sonography[Title/Abstract] OR sonographies[Title/Abstract] OR sound spectrogram*[Title/Abstract] OR vocalization recording*[Title/Abstract]
S4. 2 OR 3
S5. ambulatory[Title/Abstract] OR bracelet*[Title/Abstract] OR external[Title/Abstract] OR head mounted[Title/Abstract] OR headset*[Title/Abstract] OR head set*[Title/Abstract] OR home based[Title/Abstract] OR home environment[Title/Abstract] OR mobile[Title/Abstract] OR natural environment[Title/Abstract] OR naturalistic[Title/Abstract] OR personal[Title/Abstract] OR point of care[Title/Abstract] OR portable[Title/Abstract] OR remote[Title/Abstract] OR skin mounted[Title/Abstract] OR noninvasive[Title/Abstract] OR non invasive[Title/Abstract] OR smart[Title/Abstract] OR tetherless[Title/Abstract] OR untethered[Title/Abstract] OR wearable[Title/Abstract] OR wireless*[Title/Abstract] OR wrist mounted[Title/Abstract]
S6. "Galvanic Skin Response"[Mesh] OR "Electroencephalography"[Mesh] OR "Electrocardiography"[Mesh]
S7. biosensor*[Title/Abstract] OR brain monitor*[Title/Abstract] OR device*[Title/Abstract] OR ECG[Title/Abstract] OR EEG[Title/Abstract] OR electrocardiography[Title/Abstract] OR electrocardiogram[Title/Abstract] OR electrodermal[Title/Abstract] OR electroencephalogram[Title/Abstract] OR electroencephalography[Title/Abstract] OR FNIRS[Title/Abstract] OR functional near infrared spectroscopy[Title/Abstract] OR "heart monitor"[Title/Abstract] OR "heart monitors"[Title/Abstract] OR "heart monitoring"[Title/Abstract] OR sensor*[Title/Abstract] OR skin conductance[Title/Abstract]
S8. 6 OR 7
S9. 5 AND 8
S10. 4 OR 9
S11. 1 AND 10
S12. (animals[MeSH Terms]) NOT (humans[MeSH Terms])
S13. 11 NOT 12
S14. LIMIT TO 1990-2024
S15. LIMIT TO Language=English

***Web of Science Core Collection***

S1. TS=(( "angelman syndrome" OR "angelman's syndrome" OR "happy puppet" OR UBE3A) )
S2. TS=(( accelerometry OR accelerometer* OR actigraph* OR actigram OR electropalatograph* OR electroglottography OR “eye movement tracking” OR “eye tracking” OR “glottal waveform*” OR gyroscope* OR “hearing aid*” OR “hearing test*” OR inertia OR inertial OR “language environment analysis” OR “motion sensor*” OR “motion sensing” OR “nasal oral ratio*” OR nasometry OR nasality OR nasalance OR “physiological arousal” OR polysomnography OR polysomnographies OR “sleep monitor*” OR “smart bandage*” OR “smart device*” OR “smart glasses” OR “smart phone*” OR smartglasses OR smartphone* OR somnography OR somnographies OR sonography OR sonographies OR “sound spectrogram*” OR “vocalization recording*” ) )
S3. ( ambulatory OR bracelet* OR external OR “head mounted” OR headset* OR “head set*” OR “home based” OR “home environment” OR mobile OR “natural environment” OR naturalistic OR personal OR “point of care” OR portable OR remote OR “skin mounted” OR noninvasive OR “non invasive” OR smart OR tetherless OR untethered OR wearable OR wireless* OR “wrist mounted” )
S4. TS=( biosensor* OR “brain monitor*” OR device* OR ECG OR EEG OR electrocardiography OR electrocardiogram OR electrodermal OR electroencephalogram OR electroencephalography OR FNIRS OR functional near infrared spectroscopy OR "heart monitor*" OR sensor* OR “skin conductance” )
S5. #3 AND #4
S6. #2 OR #5
S7. #1 AND #6
S8. #7 AND PY=1990-2024
S9. #8 AND Language=English
